# Sharing sleeping sites disrupts sleep but catalyses social tolerance and coordination between groups

**DOI:** 10.1098/rspb.2024.1330

**Published:** 2024-11-06

**Authors:** J. Carter Loftus, Roi Harel, Alison M. Ashbury, Chase L. Núñez, George P. Omondi, Mathew Muttinda, Akiko Matsumoto-Oda, Lynne A. Isbell, Margaret C. Crofoot

**Affiliations:** ^1^ Department for the Ecology of Animal Societies, Max Planck Institute of Animal Behavior, Radolfzell 78467, Germany; ^2^ Department of Biology, University of Konstanz, Konstanz 78457, Germany; ^3^ Centre for the Advanced Study of Collective Behaviour, University of Konstanz, Konstanz 78457, Germany; ^4^ Department of Anthropology, University of California, Davis, CA 95616, USA; ^5^ Animal Behavior Graduate Group, University of California, Davis, CA 95616, USA; ^6^ Mpala Research Centre, Nanyuki P.O. Box 555 - 10400, Kenya; ^7^ Department of Veterinary Population Medicine, University of Minnesota, Saint Paul, MN 55108, USA; ^8^ Department of Clinical Studies, Animal Health Innovation Laboratory, University of Nairobi, Nairobi P.O. Box 30197-00100, Kenya; ^9^ Department of Veterinary and Capture Services, Kenya Wildlife Service, Nairobi P.O. Box 40241 - 00100, Kenya; ^10^ Graduate School of Tourism Sciences, University of the Ryukyus, Okinawa 903-0213, Japan

**Keywords:** social tolerance, social sleep, intergroup interactions, site sharing, night-time behaviour

## Abstract

Sleeping refuges—like other important, scarce and shareable resources—can serve as hotspots for animal interaction, shaping patterns of attraction and avoidance. Where sleeping sites are shared, individuals balance the opportunity for interaction with new social partners against their need for sleep. By expanding the network of connections within animal populations, such night-time social interactions may have important, yet largely unexplored, impacts on critical behavioural and ecological processes. Here, using GPS and tri-axial accelerometry to track the movements and sleeping patterns of wild olive baboon groups (*Papio anubis*), we show that sharing sleeping sites disrupts sleep but appears to catalyse social tolerance and coordinated movement between groups. Individual baboons experienced shorter and more fragmented sleep when groups shared a sleeping site. After sharing sleeping sites, however, otherwise independent groups showed a strong pattern of spatial attraction, moving cohesively for up to 3 days. Our findings highlight the influence of night-time social interactions on daytime social relationships and demonstrate how a population’s reliance on, and need to share, limiting resources can drive the emergence of intergroup tolerance.

## 1. Introduction

Important, scarce and shareable resources bring animals together, shaping patterns of attraction and avoidance among individuals, groups and populations, and thereby structuring the social landscape [1–3]. From forest elephants at bias to migratory birds at rubbish dumps, attraction to rare resource-rich areas often brings otherwise discrete social units into contact with one another, with important implications for biological processes such as gene flow, disease transmission and information spread [4–6]. Similarly, in habitats where safe sleeping sites are limited, night-time refuges can act as social hotspots [7–11]. Little is known about the social dynamics at sleeping sites because wild animals are rarely studied during their sleeping periods. However, by creating opportunities to interact with new social partners and forge social relationships across group boundaries, shared sleeping sites may play an important, if underappreciated, role in shaping patterns of social interaction across landscapes.

In addition to their influence on the network structure of populations, social interactions at shared sleeping sites may also have important effects on sleep quality [12]. Theory predicts that sleeping together provides anti-predator benefits in the form of increased collective vigilance and decreased likelihood of predation from simple dilution effects [13,14]. These reductions in risk may improve sleep quality by allowing individuals to sleep longer and more deeply [15,16]. However, empirical evidence suggests that sleeping in large groups can be quite disruptive [12]. Cascading sleep disruptions can emerge, where animals awaken their neighbours in a contagious process. Sharing sleeping sites also creates opportunities for affiliative and agonistic interaction across group boundaries that may impact sleep behaviour. Animals might, for example, go to sleep later, awaken during the night and/or wake up earlier than usual to engage in affiliative social interactions. Alternatively, by increasing the risk of conspecific aggression, sleeping in large groups may incentivize lighter sleep and lower awakening thresholds [12,17].

To understand how the decisions animals make about where and with whom to sleep influence the quality of their sleep, and to investigate whether the consequences of these choices extend beyond the sleep period to shape patterns of diurnal social interaction, we tracked the movement and sleep behaviour of wild olive baboon groups (*Papio anubis*) over 12 months. Olive baboons live in cohesive groups of 15–100 individuals, with relatively stable membership [18]. Females are the philopatric sex. Although neighbouring groups have extensive home range overlap, intergroup relationships are predominantly competitive and intolerant [19–21]. At night, however, when baboons seek refuge on cliffs and in trees to reduce the risk of predation, groups occasionally sleep together [22]. To quantify the effects of sharing sleeping sites on intergroup social interactions and individuals’ sleep quality, we collected GPS and accelerometry data from six adult females belonging to four different social groups, yielding a total of 1644 baboon-days of data (electronic supplementary material, table S1). We used permutation analyses to quantify group attraction and avoidance patterns, and a Bayesian analysis framework to compare intergroup interaction patterns after nights when study groups shared sleeping sites versus nights when they did not. Leveraging recent advances in non-invasive monitoring of sleep behaviour [23–25], we used accelerometry [26,27] to quantify individual baboons’ sleep patterns, and tested whether their sleep quality differed on nights when they did share versus did not share their sleeping site with another study group.

## 2. Material and methods

### (a) Data collection

We monitored the movement and activity patterns of six adult female olive baboons from four social groups that live in and around the Mpala Research Centre, a 200 km^2^ conservancy of savannah–woodland habitat located on the Laikipia Plateau in central Kenya [28]. Each study individual was trapped, anaesthetized and fit with a 450 g GPS and accelerometer collar (Savannah Tracking, Inc., Nairobi, Kenya; see [29] for details on capture methodology). We programmed the collars to record GPS locations at 15 min sampling intervals and to collect bursts of tri-axial accelerations for 3 s min^−1^ at 10.54 Hz/axis continuously throughout the study. The collars collected data from as early as 15 January 2014 to as late as 27 January 2015, with some collars ending data collection prematurely (electronic supplementary material, table S1). We processed the data to synchronize the GPS data across groups, interpolate missing fixes, correct anomalous GPS fixes and remove local GPS jitter that was likely caused by poor satellite reception (see electronic supplementary material).

### (b) Identifying home ranges and sleeping sites

**Figure 1.**
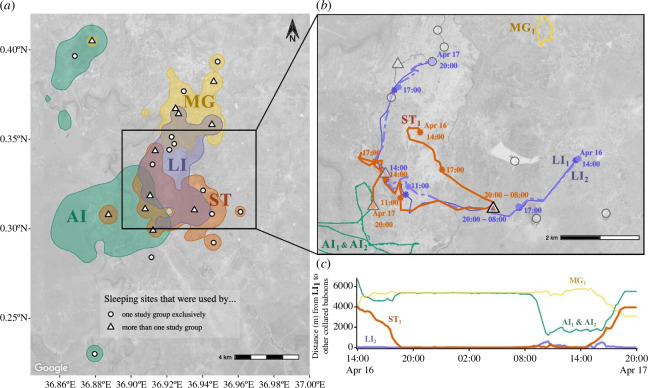
Home range overlap, shared sleeping sites and cohesive movement between interacting baboon groups. (*a*) Our four GPS-tracked study groups had highly overlapping home ranges that included 24 unique sleeping sites (points), 11 of which were used by more than one study group (triangles) during the 12 months of the study. (*b*) Out of 128 dyadic intergroup interactions, 31 involved cohesive movement between groups, as in the example depicted here: the purple group (represented by two collared individuals; the solid purple line and the dashed purple line) moved cohesively with the orange group (represented by a single collared individual, the solid orange line) after spending the night at the same sleeping site (black-outlined triangle with purple and orange fill). The concurrent movement of baboons in other groups that were not part of this interaction is depicted by green and yellow lines. (*c*) During this example interaction, the intergroup distances between the purple and orange groups (orange line) were similar to the inter-individual distances within the purple group (purple line) and shorter than the intergroup distances with the other groups (green and yellow lines). Group LI's tracking period ended after six months; however, the home ranges were similar across the study period (see electronic supplementary material, figure S7 in the electronic supplementary material for home ranges of just the first six months of the study).

We created group-level tracks based on the tracks of collared females(figure 1). The average distance between two individuals (dyadic distance) of females belonging to the same group was 43.4 ± 0.3 m (mean ± s.e.), falling within the typical range of baboon group spread (75–350 m [30]). Data from a single group member were used to describe the location of the group. We removed group-level duplicate data (i.e. data on two females belonging to the same group) when data were sampled simultaneously. In these cases, we excluded the data from the female in the group that had the shortest duration of data collection. From a single individual per group, we calculated the utilization distributions (UDs) for each group using kernel density estimation. We define each group’s home range as the minimum boundary encompassing 95% of the UD. For each group dyad, we calculated the proportion of overlap of the groups’ home ranges (electronic supplementary material, table S2), as well as the Bhattacharyya’s affinity between their UDs for a more accurate probability of the groups’ joint space use ([30]; electronic supplementary material, table S3). For all home range analyses, we used the adehabitatHR package [31] for R v. 4.1.2 [32].

We determined where each individual spent each night by calculating the mean location of her GPS fixes from 20.00 to 04.00. With hierarchical agglomerative clustering (stats package, [33]) of the mean night-time locations, we identified 24 distinct sleeping sites used by the study population. The estimated area of the clusters was 166 ± 24 m^2^ and the distance between clusters was 6084 ± 198 m (mean ± s.e.). We used the cluster membership of individuals’ mean night-time locations to determine which sleeping site they took refuge in each night. Sleeping sites were considered ‘shared’ when more than one group spent the night at the same site.

### (c) Inferring the spatial scale of interaction

**Figure 2.**
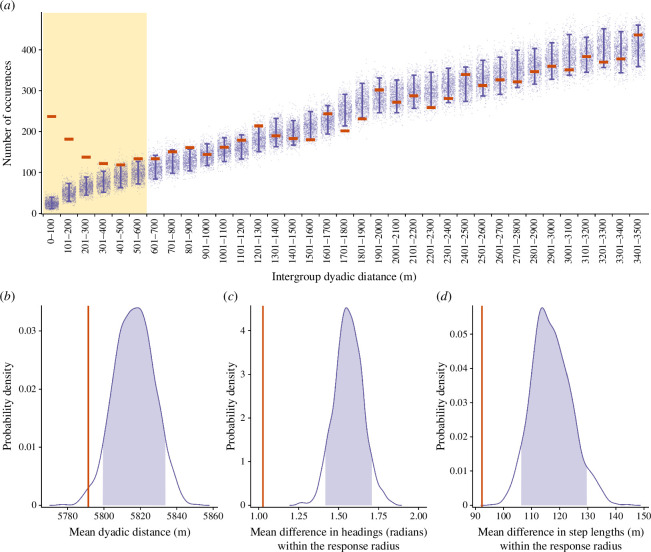
Inferring the spatial scale of interaction and characterizing patterns of proximity and movement between baboon groups. (*a*) Because the 601–700 m distance bin is the first (when ascending from zero) in which the empirical value (horizontal orange lines) falls within the 5th and 95th percentile of the corresponding null distribution (purple error bars summarizing the purple dots, i.e. null model), we determined that baboon groups probably detect and respond to neighbouring groups at distances up to 600 m (i.e. the ‘response radius’), indicated here with a yellow-shaded background. Within the response radius, it is likely that individuals from neighbouring groups could detect each other via visual or acoustic cues in this habitat. For visualization purposes, the *x*-axis only extends to 3500 m (for the full plot, see electronic supplementary material, figure S3). (*b*) Across the entire dataset, baboon groups maintained shorter pairwise intergroup distances than expected by chance. While they were within the 600 m response radius, interacting baboon groups had (*c*) a smaller mean difference in headings and (*d*) a smaller mean difference in step lengths than expected by chance. In (*b–d*), the means of the empirical data are represented by the vertical orange lines, while the distribution of means from the null model is represented by the purple lines, with the 90% inter-quantile areas shaded in purple.

We developed an algorithm to empirically extract the ‘response radius’ (i.e. the spatial threshold at which intergroup proximity translated into detectable changes in movement) from the GPS data (figure 2*a*; see electronic supplementary material for further details). We calculated the Euclidean distance between every study group dyad at each time stamp and defined dyadic intergroup interactions as periods when a collared member of a group was within the response radius of a collared member of another group (between 8.30 and 17.30, the median departure and arrival times at sleeping sites, respectively; electronic supplementary material, figure S1). Interactions between a group dyad that were less than 75 min apart were grouped together (and are henceforth considered single interactions). When groups interacted during one day, shared a sleeping site and then interacted on the next day (i.e. continuously remaining within the response radius), this was counted as two interactions—one on the first day and another one on the second day, with sleeping site sharing in between. Groups were classified as moving cohesively when two collared individuals from different groups moved at least 100 m while remaining within the 80% quantile of the daytime inter-individual distances between collared group mates (electronic supplementary material. figure S2), i.e. the movement of collared individuals in the different interacting groups was indistinguishable from the movement of collared group mates. During all encounters, we tested whether, within a dyad, a particular group tended to be in front of or behind the other group by comparing the distances between each group at each time step and the centroid of the two groups at the next time step—i.e. with respect to the direction of travel, which group was in front and which was behind. Positive values represent cases in which the first group in the dyad is in the front, and negative values represent cases in which the second group is in the front. A zero value means that the groups were even with respect to the direction of travel.

### (d) Movement coordination and occupancy of sleeping sites

To better understand the nature of intergroup relationships, we used permutation analyses to investigate interdependencies in the movements and sleeping site choices of groups, while controlling for attraction to shared resources. We took movement trajectories of each group dyads and randomly shifted the days of one group, for 1000 permutations—essentially creating 1000 new pairwise datasets wherein all properties of the locations and movement trajectories of the group were preserved except for their concurrent timing. These new datasets produced a null distribution of intergroup movement and space-use characteristics (e.g. the dyadic distances between groups) that could have occurred by ‘chance’, i.e. if the groups were moving independently of one another [34]. We then compared the empirical values (from the real dataset) of particular movement and space-use characteristics with the distributions of values from the null model: where an empirical measure fell outside the 90% inter-quantile distance of the null distribution, we determined that characteristic to be significantly different from what would be expected by chance, and thus likely to have been influenced by social forces between two groups. We then compared intergroup movement metrics in the empirical data with the distributions of these same metrics derived from the null models. In total, we used this basic principle to create three null models.

1. *Attraction/avoidance*. We first tested whether groups avoided or were attracted to each other by measuring the mean dyadic distance between each group dyad, as well as the proportion of time that dyads spent in association (i.e. within the 600 m response radius) (figure 2*a*), and comparing these metrics between the empirical data and the null model. To understand how behaviour within the response radius compared to that expected by chance, we took the same approach, calculating and comparing (i) the mean distance between the two groups, (ii) the mean difference between the headings of the groups, and (iii) the mean difference between the groups' step lengths (e.g. displacements between each 15 min interval).
2. *Sleeping site choice*. We assessed whether groups influenced each other’s sleeping site choices by determining whether groups shared the same night-time refuge more or less often than expected by chance. Specifically, we compared the probability of our study groups sharing a sleeping site in the empirical data and in the permuted null model.
3. *Daytime movement after leaving a shared sleeping site*. We assessed whether groups stayed closer together and showed more similar movement trajectories after sharing a sleeping site than expected by chance. We generated site-specific null models by subsetting only the days after groups had ‘shared’ each sleeping site from the permuted data. We then compared each of these null models to the empirical daytime movement after groups had actually shared the specific sleeping site, looking at (i) the mean distance between the two groups, (ii) the mean difference between the headings of the groups when inside the response radius, and (iii) the mean difference between the step lengths of the groups when inside the response radius. In this analysis, we focus on only two sleeping sites because they were the only two for which there were enough instances of ‘sharing’ in the permuted data to generate a reliable null model.

### (e) Sleep analysis

We extracted sleep metrics from the accelerometry data with an adaptation of the algorithms presented by van Hees and colleagues [35,36] that were developed for analysing sleep in humans using wearable accelerometry devices. Our adaptation of the sleep classification algorithm, which was implemented and validated in [12], exhibited 80.7% accuracy in distinguishing sleep and waking behaviour in wild olive baboons. Using the accelerometer-based sleep classification algorithm, we calculated the total sleep time as the total number of sleep epochs between 21.00 and 05.00 (as this time period consistently fell within the bounds of the sleep period of all individuals); sleep efficiency as the proportion of epochs during the sleep period that were classified as sleep; and sleep fragmentation as the number of distinct awake bouts during the sleep period that were greater 2 min or longer in duration, divided by the total number of hours of sleep during the sleep period [37]. We also measured sleep synchronization as the number of minutes during which each dyad exhibited the same behaviour (either sleeping or awake) divided by the total number of minutes for which we had accelerometry data (and thus, sleep behaviour data) for both individuals (see electronic supplementary materials for details of the sleep classification algorithm and detailed definitions of sleep metrics).

### (f) Statistical analysis

For all comparisons of empirical data versus null models, we used Fisher’s exact test. The *p*-value, therefore, represents the proportion of values from the null distribution that are as extreme or more extreme than the empirical value.

To test how sharing a sleeping site influenced intergroup interactions during the following day, we subset the data to days during which two groups interacted with each other, and then modelled the probability that the groups exhibited cohesive and coordinated movement on a given day (family = Bernoulli) and the total time that the two groups spent interacting (family = Gamma), as a function of whether the two groups had slept at the same sleeping site on the previous night. For both models, we used hierarchical Bayesian regression, with random intercept terms for the identity of each group involved in the encounter and the identity of the group dyad.

We tested the influence of sharing a sleeping site with another group on the total sleep time, sleep efficiency and sleep fragmentation. We modelled the total sleep time while at the sleeping site using a hierarchical Bayesian regression model (family = Student’s *t*), with a fixed effect variable indicating whether the group was sharing its sleeping site on that night with another group, and random intercept terms for group identity, individual identity, date and the identity of the sleeping site. By including the identity of the sleeping site as a random effect, we controlled for variation among the sleeping sites in their characteristics that may inherently promote or hinder high-quality sleep. We used the same fixed and random effect variables to model the log odds of sleep efficiency, as well as sleep fragmentation, both with hierarchical Bayesian regression. We then tested whether individuals from different groups were more likely to show synchronization in their night-time sleep/wake patterns when they shared the same sleeping site. We modelled the synchronization score (see the electronic supplementary material) between each dyad as a function of whether dyad members were sharing the same sleeping site, using hierarchical Bayesian regression. We included random intercepts for the identity of both members of the dyad, as well as for the identity of the dyad itself.

We fit all Bayesian models with the *brms* package in R [38]. We used diffuse, mean-zero Gaussian priors for fixed effects variables. For random effects variables and the intercept, we used half Student’s *t* distributions with three degrees of freedom and a scale parameter of 2.5 as prior distributions. Model estimates are based on four independent Hamiltonian Monte Carlo chains with 2500 burn-in iterations and 2500 sampling iterations. Trace plots indicated adequate mixing and convergence of the four chains on the same posterior region. In the main text, we report the mean of the posterior distribution, along with the lower and upper 90% credible interval bounds from the standardized models for all model estimates. The model output tables were generated using *sjPlot* [39] and can be found in the electronic supplementary material.

### (g) Data visualization

All plots were generated in R using the *ggplot2* [40] and *cowplot* [41] packages. Additionally, the map figures were generated using the *ggmap* [42] and *sf* [43] packages, and the half violin plots were generated using the *gghalves* [44] package.

## 3. Results

Baboons in this population showed evidence of spatial attraction among social groups. In addition to sharing substantial portions of their home ranges (figure 1, electronic supplementary material, tables S2, S3), study groups were, on average, significantly closer to one another than expected by chance (figure 2*b*; *p *< 0.01). Using a permutation paradigm, we found that the observed patterns of intergroup spatial proximity diverged from the expectations of a null model at distances of 600 m (figure 2*a*)—henceforth, the response radius. Within this response radius, groups’ movement trajectories showed evidence of coordination, with headings (figure 2*c*) and step lengths (figure 2*d*) more similar than expected based on our permuted null models (Fisher’s exact tests: headings: *p *< 0.0001, step lengths: *p *< 0.0001).

**Figure 3.**
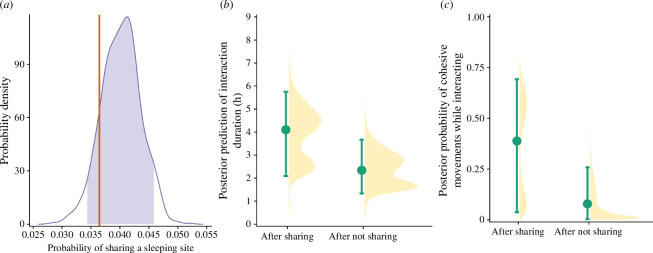
The influence of sharing sleeping sites on daytime interactions. (*a*) The probability of baboon study groups sharing a sleeping site was well within the range expected by chance. The empirical data are represented by the vertical orange line, while the distribution of means from the null model is represented by the purple distribution, with the 90% inter-quantile area shaded in purple. Given that two groups did interact, (*b*) the posterior prediction of the total time (in hours) that the two groups spent interacting after they had shared a sleeping site was greater than after they had not shared a sleeping site and (*c*) the posterior probability that the two groups moved cohesively while interacting after they had shared a sleeping site was greater than after they had not shared a sleeping site. In (*b*) and (*c*), shaded yellow areas show the full posterior distributions, green points depict the means and the green whiskers extend to the minimum and maximum of the 90% credible interval.

Patterns of spatial attraction between study groups were driven by their sharing of sleeping sites. Dyadic distances between neighbouring baboon groups were significantly smaller than expected by chance on days following sleeping site sharing (*p *< 0.0001), but the same was not true when groups slept at separate sites on the preceding night (*p* = 0.253). By definition, interactions are more likely to occur when groups start the day at the same location. Indeed, study groups interacted during 95.7% of days (44/46) that followed nights when they slept together, whereas they interacted on just 4.6% of days when they had slept separately the preceding night (66/1436 group-dyad days). However, the sharing of sleeping sites was also associated with changes in the duration and tenor of intergroup interactions. Given that an interaction occurred, study groups spent more time together (figure 3*b*) and were 4.9 times more likely to move cohesively (figure 3*c*) on days after they had shared a sleeping site compared with days after they slept separately (time spent interacting after sharing a sleeping site: 3.90 [2.08, 5.73] h; time spent interacting after not sharing a sleeping site: 2.37 [1.32, 3.68] h; probability of cohesive movement after sharing a sleeping site: 0.40 [0.04, 0.70] and probability of cohesive movement after not sharing a sleeping site: 0.07 [0, 0.26]). Positioning during coordinated movement bouts was not consistent with groups herding one another: neither group was consistently in front or behind the other with respect to their travel direction (electronic supplementary material, figure S4). After groups shared a sleeping site, periods of coordinated movement did not arise solely because the physical structure of shared sleeping sites and their environs constrained baboons’ movement choices. In the days after sharing a sleeping site, group dyads travelled in closer proximity, for longer periods of time and with more closely aligned headings, than by chance, based on a site-specific null model designed to control for such ‘environmental forcing’ (electronic supplementary material, figure S5).

Baboon sleeping sites are limited on the landscape: the four groups in this study slept at only 24 distinct locations during the study period. All sleeping sites were situated on cliff faces (13/24) or in acacia tree groves (11/24) (for details, see [22]) and nearly half were used by more than one study group during the data collection period (11/24; figure 1*a*). However, baboon groups did not coordinate their sleeping site selection: groups shared sleeping sites on 3.36 ± 1.30% of the nights when they were tracked (mean ± s.e. across the four groups), a rate that is statistically indistinguishable from chance (figure 3*a*; Fisher’s exact test: *p* = 0.291).

**Figure 4.**
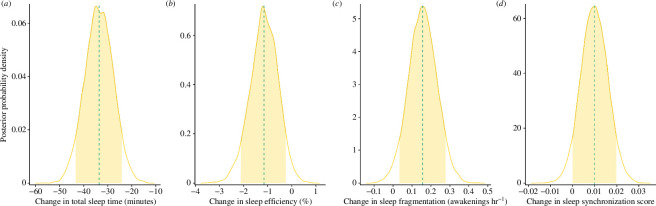
The effects of sharing sleeping sites on baboons’ sleep quality. Baboons had (*a*) shorter total sleep times, (*b*) lower sleep efficiency, (*c*) higher sleep fragmentation and (*d*) higher inter-individual sleep synchronization, when groups shared sleeping sites versus when they did not. In all plots, the distribution represents the change in the posterior probability densities between nights when they had shared a sleeping site and nights when they had not shared a sleeping site. The shaded yellow areas are the 90% credible intervals and the dotted green lines depict the means.

Despite this apparent indifference, sharing a sleeping site with a neighbouring group negatively impacted baboons’ sleep quality. Using an algorithm to identify bouts of sleep from accelerometry data [12], we found that baboons slept for 9.3 ± 0.03 h (median ± s.e.) per night. Overall, individuals experienced sleep onset at 19.32 ± 2.4 min and awoke at 05.55 ± 2.7 min. Within this sleep period, baboons’ sleep efficiency was 81.4 ± 0.1% and they averaged 2.0 ± 0.02 awakenings per hour of sleep. However, on the subset of nights when baboons shared their sleeping site with another group, they slept for 33.5 fewer minutes (figure 4*a*), experienced a 1.2% reduction in sleep efficiency (figure 4*b*) and experienced 0.15 more awakenings per hour of sleep (figure 4*c*; total sleep time −0.52[−0.67,−0.37]; log-odds of sleep efficiency: −0.20 [−0.35,−0.04]; sleep fragmentation: 0.20 [0.05, 0.35]). Baboons from different groups also showed greater synchronization in their sleep patterns during the night when they shared a sleeping site compared to when they slept at separate sites (synchronization score: 0.18 [0.00, 0.37]; figure 4*d*).

## 4. Discussion

To satisfy their need for sleep, all animals must make decisions about when, where and with whom they can safely enter this vulnerable state [15,45]. These choices are consequential, yet behavioural research focuses almost exclusively on what animals do during their waking periods and researchers have largely ignored animals’ sleep periods and the potential consequences of their night-time decisions. Leveraging technological advances to remotely monitor baboon movement and sleep behaviour, we show a pattern of intergroup attraction in our study population that is precipitated by mutual attraction to night-time refuges—important, scarce and yet shareable resources. Although baboons experienced reduced sleep quality when groups slept together, sharing of sleeping sites was associated with prolonged and apparently tolerant interactions between the groups on the subsequent day.

Considerable social diversity exists within the genus *Papio*, including differences in the structure of social relationships between groups [46]. While neighbouring groups have overlapping home ranges in all six baboon species, the relationships between groups vary from mutually intolerant or avoidant (yellow baboons, *P. cynocephalus* [20,21] and chacma baboons, *P. ursinus* [47]) to tolerant or integrated into multi-level societies (Guinea babooons, *P. papio* and hamadryas baboons, *P. hamadryas* [48,49]). Similar to yellow and chacma baboons, the intergroup relationships of olive baboons—our study species, *P. anubis*—have been described as competitive in tenor despite significant home range overlap [50,51]. Our finding that groups are, on average, in closer proximity than expected by chance and that they engage in prolonged, cohesive interactions involving coordinated movement is, therefore, surprising (and stands in contrast to what has been found in yellow baboons [20]). The spatial attraction between groups that we found cannot be attributed to mutual attraction to shared resources (such as feeding trees or water sources) or constraints on travel routes driven by environmental and topographical features (such as rivers or canyons), as our analytical approach accounts for these; by permuting the real data to build null models, we ensure that the relationship between baboons’ movement paths and their resource and physical landscape is preserved. Instead, the attraction between groups appears to be driven by changes in the tenor of social interactions between groups after sharing sleeping sites.

Baboons in this study showed neither a preference for, nor an avoidance of, sharing sleeping sites; groups slept together at the same location at rates expected by chance. Although groups did not sleep together often—sharing of sleeping sites was only observed on 3% of nights—these events played an outsized role in shaping patterns of interaction between groups in this population (electronic supplementary material, figure S8). Nearly 40% of all intergroup interactions occurred on a day that followed sharing a sleeping site. Baboon groups behaved differently during these interactions as compared to those that took place when groups had not slept together on the preceding night: interactions were, on average, nearly twice as long and groups were more than four times as likely to travel cohesively. During these periods of cohesive movement, the dyadic distances and alignment of travel trajectories of tracked individuals in different groups were indistinguishable from those of members of the same group, indicating that groups were coordinating their movement. This coordination was not simply a result of constraints imposed by the environment. We did not observe this high cohesion and alignment in null models where we compared the trajectories of groups departing from the same sleeping site, but on different days (i.e. where the environmental constraints were the same but the social context was removed). Observed patterns of coordinated movement were also unlikely to be the result of one group herding or chasing the other. Although our GPS tracking data, with one point every 15 min, are insufficient to resolve such fine-scale behaviour, the extended nature of these interactions and the fact that, within a bout of coordinated movement, neither group was consistently positioned in front of (or behind) the other, does not support the interpretation that coordinated movement is being driven by competitive or agonistic social dynamics. It is, of course, also possible that an unmeasured variable explains both the sharing of sleeping sites and coordinated movement during the following day; for example, it is plausible that the presence of a predator may lead groups to sleep and move together.

On nights when baboons shared sleeping sites, members of co-sleeping groups showed synchronicity in their wake/sleep patterns. This result is contrary to the predictions of the sentinel hypothesis [52,53], which suggests that co-sleeping animals can gain anti-predator benefits by desynchronizing their wakening periods so as to maximize group vigilance. It is, however, consistent with our previous findings on the social dynamics of sleep in baboons [12], and is, to a large degree, unsurprising. Individuals sleeping in the same location experience a shared environment and probably awaken in response to the same night-time disturbances. Thermal recordings of other baboon groups at their sleeping sites reveal considerable night-time activity [JC Loftus, R Harel and MC Crofoot, 2019, unpublished data], leading us to hypothesize that a complementary social dynamic is also at play: night-time interactions across group boundaries may enhance patterns of wake/sleep synchronicity and could explain the social tolerance and between-group coordination we observed following the sharing of sleeping sites.

Baboons may derive several benefits from associating with members of other groups at night that would be harder to achieve during typical daytime encounters, including mating outside their group, extending their social networks and investigating emigration options. This is, in part, because social interactions between groups may be inherently less competitive during the night than in the daytime because baboons are not competing for food resources [54–56]. Furthermore, poor night vision in conjunction with the increased number of individuals at shared sleeping sites may limit baboons’ ability to monitor their group mates—allowing certain individuals more freedom for between-, but also within-group social exploration [21]. While further research will be needed to understand the trade-offs that animals face when sleeping in a social context, our results suggest that the influence of night-time social dynamics can extend well beyond the sleep period, and may even shape the structure of a population’s social connections.

Regardless of the potential social benefits derived from sharing sleeping sites, our results document that these come at a cost to sleep quality: baboons slept less, experienced lower sleep efficiency and had more fragmented sleep when spending the night with another group. This conflicts with the predictions of existing hypotheses about social sleep—namely that individuals should sleep better when sharing a sleeping site owing to the anti-predator benefits of increased vigilance and increased predator dilution [15]. Instead, diminished sleep quality may reflect an incidental cost of chance encounters at the sleeping site, perhaps arising from the stress induced by intergroup interactions [57,58], a shortage of high-quality sleep positions within the sleeping site that can accommodate the greater number of individuals [17], and/or social interactions or disruptions [12,59]. Our analyses probably underestimate the true degree of sleep disruption from sharing sleeping sites because untracked baboon groups range in our study area, meaning that study groups were not necessarily alone on nights when they did not share a sleeping site with another study group. In humans, even mild sleep loss leads to quantifiable performance deficits [60], suggesting that the effects we document—including a roughly 30 min reduction in sleep time when sleeping with another group—may have important behavioural, cognitive and physiological ramifications for baboons. On the other hand, recent studies of sleep in wild animal populations have documented unexpected plasticity in sleep needs in some species, introducing the concept of ‘adaptive sleep loss’ [16]. When multiple baboon groups share a sleeping site, it is possible that the associated reduction in sleep quality is either less costly than we might anticipate based on the human sleep literature and/or that these costs may be (at least partially) offset by other benefits gained through night-time interaction.

To the best of our knowledge, all animals sleep [61], yet we still know surprisingly little about the sleep of non-model organisms, especially in ecologically relevant field contexts. Non-invasive approaches to sleep monitoring, including accelerometry, are making it possible to monitor and quantify the sleep patterns of wild animals [23,62]. Night-vision and thermal imaging technologies can provide much needed social and environmental context to such data, making it possible to investigate—in detail—the causes of night-time sleep disruptions and determine the nature of night-time social interactions. These technologies allow researchers to ‘observe’ what was previously not observable, and thus largely ignored: the night-time behaviour of diurnal animals in the wild. Future research using this approach will reveal the specific causal mechanisms that link the sharing of sleeping sites with daytime social interactions among baboons and, in so doing, will provide a more holistic understanding of how social decisions made during the active period shape, and are shaped by, those made at night.

Safe night-time refuges are critical, yet limiting, resources for many animals [20,63]. By bringing conspecifics together in time and space, sleeping sites become social hotspots, creating opportunities for otherwise distinct social units to interact. Social relationships forged or reinforced at shared sleeping sites appear critical to the maintenance of multi-level social structures in many primate species [49,64–68]. Although olive baboons do not show the highly modular social structure typical of multi-level societies in primates, this study suggests that neighbouring groups do form differentiated social relationships that, particularly in the context of shared sleeping sites, can be tolerant and, perhaps, even affiliative. These social dynamics are reminiscent of the form of multi-level sociality that has recently been reported in vulturine guineafowl that live in the same habitat [11] and suggest that higher-order networks [69] may emerge spontaneously in populations of group-living animals whose members rely on, and are generally tolerant at important, scarce and shareable resources. A better understanding of how environmental features shape the social landscape is a priority because such higher-order structuring of a population’s social network has important implications for key biological processes [70,71]. Taking advantage of technological advances, future behavioural research should aim to widen our field of view from individuals and groups to the networks of social interaction that structure neighbourhoods and populations.

## Data Availability

The datasets analyzed in this study are available in the Movebank Data Repository, https://doi.org/10.5441/001/1.625 [28]. All code used to produce the final results from the raw data is on GitHub (https://github.com/CarterLoftus/intergroup_sleep) and will be archived on Zenodo. Supplementary material is available online [72].
